# P-341. Multidrug-Resistant Organism Sharing Among Nursing Home Roommates

**DOI:** 10.1093/ofid/ofae631.543

**Published:** 2025-01-29

**Authors:** Gabrielle Gussin, Ken Kleinman, Raveena D Singh, Justina Bui, Gabriel Gadia, Thomas T Tjoa, John Mitchell, Raheeb Saavedra, Julie A Shimabukuro, Cassiana E Bittencourt, Susan Huang

**Affiliations:** University of California, Irvine School of Medicine, Division of Infectious Diseases, Irvine, California; University of Massachusetts Amherst, Amherst, Massachusetts; University of California, Irvine School of Medicine, Division of Infectious Diseases, Irvine, California; University of California, Irvine, Irvine, California; University of California, Irvine, Irvine, California; University of California, Irvine School of Medicine, Division of Infectious Diseases, Irvine, California; University of California, Irvine, Irvine, California; University of California, Irvine School of Medicine, Division of Infectious Diseases, Irvine, California; University of California, Irvine Health, Orange, California; University of California, Irvine Health, Orange, California; University of California, Irvine School of Medicine, Irvine, California

## Abstract

**Background:**

Nursing homes are high-risk settings for multidrug-resistant organism (MDRO) prevalence and spread. We investigated whether nursing home roommates were more likely to carry the same MDRO compared to non-roommates.

**Figure:**
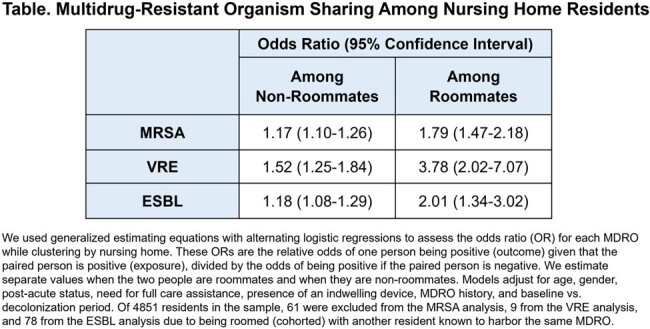

**Methods:**

We conducted a secondary analysis of 44 nursing homes in two studies (Protect Trial-Miller NEJM 2023; SHIELD-Gussin JAMA 2024) involving universal chlorhexidine bathing and nasal iodophor. We used MDRO status from baseline (2016-17) and end-intervention (2018-19) visits where 50 residents were sampled at random at each nursing home. Sampling included bilateral nares swabs processed for methicillin-resistant *Staphylococcus aureus* (MRSA), and axilla/groin swabs processed for MRSA, vancomycin-resistant *Enterococci* (VRE), and extended-spectrum beta-lactamase (ESBL) producers.

We used generalized estimating equations with alternating logistic regressions to assess the odds ratio (OR) for each MDRO while clustering by nursing home. These ORs are the relative odds of one person being positive (outcome) given that the paired person is positive (exposure), divided by the odds of being positive if the paired person is negative. We estimated separate values when the two people are roommates and when they are non-roommates.

Models adjusted for age, gender, post-acute status, need for full care assistance, presence of an indwelling device, MDRO history, and baseline vs. decolonization period. Shared room assignments (cohorting) due to known history of the specific MDRO being modeled were excluded.

**Results:**

The 44 nursing home sample included 4851 residents who were roomed in 180 single rooms, 1945 doubles, 874 triples, 93 quads, four 5-bed rooms, and six 6-bed rooms. The Table shows significantly higher ORs of concordant MDRO carriage among roommates than among non-roommates for each MDRO.

**Conclusion:**

Roommates in nursing homes are significantly more likely to carry the same MDRO versus non-roommates, indicating potential transmission within shared rooms.

**Disclosures:**

**Ken Kleinman, ScD**, Xttrium Laboratories: Conducting studies in which participating hospital patients received contributed antiseptic products outside the submitted work **Raveena D. Singh, MA**, Xttrium Laboratories: Conducting studies in which participating hospital patients received contributed antiseptic products outside the submitted work **Raheeb Saavedra, AS**, Xttrium Laboratories: Conducting studies in which participating hospital patients received contributed antiseptic products outside the submitted work **Susan Huang, MD, MPH**, Xttrium Laboratories: Conducting studies in which participating hospital patients received contributed antiseptic products outside the submitted work

